# Postoperative clinical and functional outcomes in patients with tumor and tumor-like lesion of foot and ankle

**DOI:** 10.1186/s13047-022-00582-z

**Published:** 2022-10-14

**Authors:** Yasuhiko Kokubu, Toshifumi Fujiwara, Koh Nakagawa, Nokitaka Setsu, Makoto Endo, Jun-Ichi Fukushi, Yoshihiro Matsumoto, Yasuharu Nakashima

**Affiliations:** 1 Department of Orthopaedic Surgery, Graduate School of Medical Sciences, Kyushu University, 3-1-1 Maidashi, Higashi-ku, Fukuoka, 812-8582 Japan; 2 Department of Orthopaedic Surgery, National Hospital Organization Kyushu Cancer Center, 3-1-1 Notame, Minami-ku, Fukuoka, 811-1395 Japan; 3 Department of Orthopedics Surgery and Rheumatology, National Hospital Organization Kyushu Medical Center, 1-8-1 Jigyohama, Chuoh-ku, Fukuoka, 810-0064 Japan

**Keywords:** Foot and ankle, Tumor, JSSF scales, Clinical outcomes

## Abstract

**Background:**

Tumors and tumor-like lesions of the foot and ankle are relatively rare and their postoperative clinical outcome has not been well reported.

**Methods:**

This study retrospectively reviewed medical records of all patients who underwent excision of tumors and tumor-like lesions of the foot and ankle from 2008 to 2020. Preoperative and postoperative clinical outcomes were evaluated by the Japanese Society for Surgery of the Foot (JSSF) scales (pain, function, and alignment).

**Results:**

A total of 117 consecutive patients were analyzed in this study. Bone lesions accounted for 51 patients (benign: 45, intermediate malignancy: 1, malignant: 5), and soft tissue lesions accounted for 66 patients (benign: 57, intermediate malignancy: 2, malignant: 7). Four patients (8%) presenting with bone tumor and six (9%) soft tissue tumors resulted in recurrence. Eight (67%) patients with malignant lesions were alive continuously disease free and followed for a median of 50.5 (range: 18 to 82) months. Amputation at the first operation was done for five cases (33%) of malignant or intermediate malignancy (below-knee amputation: 1, Chopart disarticulation: 1, forefoot amputation: 3). Postoperative JSSF scores resulted in a significant 'positive' increase (bone lesion, 75.9 ± 13.7 to 91.4 ± 14.9, *p* < 0.001; soft tissue lesion, 84.7 ± 14.8 to 91.9 ± 12.5, *p* < 0.001). The score improvement in bone lesions was significantly higher than in soft tissue lesions (*p* = 0.003).

**Conclusion:**

The surgical management of tumors and tumor-like lesions of the foot and ankle showed good post-operative functional outcomes with bone lesions exhibiting better results when compared to soft-tissue lesions.

## Introduction

Tumors of the foot and ankle are rare entities comprising only 4 to 5% of all musculoskeletal tumors. Primary malignant tumors account for 2% of sarcomas, which make up for 0.2% of all tumors [1, 2]. In contrast, benign tumors (neurogenic tumors, lipomas, hemangiomas, and tenosynovial giant cell tumors [TGCT]) and tumor-like lesions (ganglion, synovitis, bursitis, and epidermoid cyst) occur more frequently [3]. These lesions commonly present as pain, swelling, or restricted joint range of motion, often resulting in a delayed diagnosis [4, 5]. The primary approach for management is marginal or wide excision of the tumor or the tumor-like lesion for diagnosis, pain relief, and treatment. Where patients have benign lesions after consideration by magnetic resonance imaging (MRI), or have no symptoms of dysfunction, conservative treatment is commonly selected [6, 7].

Several studies have described the distribution pattern of foot and ankle tumors [8, 9]. Bone tumors tend to occur predominantly in young males, while soft-tissue tumors are observed more commonly in females. Regarding the site of origin of the primary tumor around the foot and ankle, all four anatomic compartments, namely forefoot, midfoot, hindfoot, and ankle, are equally affected [9–11]. The foot and ankle complex has an intricate structure comprising four independent bony compartments and multiple critical functional units, including nerves, blood vessels, tendons, and ligaments, linked closely without fascial barriers. Therefore, complete surgical resection of a tumor or tumor-like lesion from this region, while maintaining the functionality, is arduous [6]. Additional concerns around an excision procedure are the integrity of the surrounding muscles and tendons and the postoperative scar, which may lead to postoperative pain and dysfunction. However, there is scarce evidence elucidating the clinical and functional outcomes after surgical excision of the foot and ankle tumors.

Therefore, the present study aimed to evaluate the postoperative clinical and functional outcomes of 117 cases of tumors and tumor-like lesions located in the foot and ankle using the Japanese Society for Surgery of the Foot (JSSF) scale. Since the JSSF scale is a modified version of the American Orthopaedic Foot and Ankle Society (AOFAS) clinical rating system [12] for the Japanese people, this study demonstrated the clinical and functional recovery after tumors and tumor-like lesions of the foot and ankle in Japan [13, 14].

## Materials and methods

### Study participants

Using a retrospective study design, we reviewed the medical records of all consecutive patients undergoing excision surgery for tumors or tumor-like lesions of the foot and ankle at Kyushu university hospital between January 1, 2008, and December 31, 2020. Clinical data of the patients, including age at the time of operation, sex, histological diagnosis–malignant or benign, site and size of the tumor, the surgical procedure conducted, recurrence, postoperative complications, and tumor outcome, were extracted. Records of patients with malignant tumors were reviewed for details of reconstruction, advanced treatment, additional surgery, and tumor prognosis. The follow-up period was calculated based on the last recorded follow-up visit date. The anatomical location of the tumor was classified according to the JSSF standard rating system as the ankle-hindfoot, midfoot, hallux metatarsophalangeal-interphalangeal, and lesser metatarsophalangeal-interphalangeal sites [13, 14]; the ankle was defined from the talocrural articulation to the same length as the widest part of the growth plate [9]. Each tumor identified on T2-weighted fat-saturated MRI was measured in millimeters, and the tumor size was defined by the greatest diameter [10]. After a full review, the authors obtained approval from the Ethics Committee of Kyushu university hospital (approval number: 26–224).

### Surgical procedure

The benign lesion was treated with marginal resection while conserving the surrounding tissue or bone curettage of the tumor with osseous transplantation. A malignant tumor was excised with a sufficiently wide margin of the surrounding normal tissue, or amputation was done [15, 16].

### Outcome measurement

Patients were categorized into two groups: bone lesions or soft-tissue lesions, and clinical outcomes were described. The preoperative and postoperative clinical outcomes were evaluated by the JSSF ankle/hindfoot, midfoot, hallux, and lesser toe scales, which consisted of three categories–pain (40 points), function (ankle/hindfoot–50 points, midfoot–45 points, hallux–45 points, and lesser toe–45 points), and alignment (ankle/hindfoot–10 points, midfoot–15 points, hallux–15 points, and lesser toes–15 points). The total score of each category was set to 100 points [13, 14, 17]. In the case of infants unable to verbally express their symptoms, clinical evaluation was conducted through detailed physical examinations and complaints highlighted by their parents.

### Statistical analysis

Mean, range, median, and rate were presented for all continuous and categorical variables. The gender distribution for tumors was compared using the Chi-square test, whereas age, maximum tumor diameter, and follow-up period were compared using the Mann–Whitney U test. The JSSF scale scores for bone and soft tissue tumors were compared using the Mann–Whitney U test, and the preoperative and postoperative JSSF scores were compared using the Student’s paired t-test. All statistical analyses were performed using the JMP software (version 14.0.0, SAS Institute Inc., Cary, NC, USA), and a *p*-value of < 0.05 was considered statistically significant. Data are given as mean ± standard deviation.

## Results

### Baseline characteristic of all participants

This study analyzed 117 consecutive patients with tumors and tumor-like lesions of the foot and ankle (Table 1), having a mean age of 33.7 years (range: 1–86 years; median: 31 years) at the time of operation. Of these 117 patients, 55 (47%) were males, and 62 (53%) were females. The mean follow-up duration was 22.2 months (range: 1–144 months, median: 10 months). Benign tumor and tumor-like lesions were observed in 102 patients (87%), intermediate malignancy in three (3%), and malignant tumors were seen in 12 patients (10%). Bone lesions were seen in 51 cases (44%) (30 males, 21 females), while soft tissue lesions were present in 66 cases (56%) (25 males, 41 females). The patients with bone lesions were younger than those with soft tissue lesions (mean age: 25.2 years vs 40.3 years), as previously reported [7].

**Table 1:** Demographic data of tumor and tumor-like lesions (*N*=117 lesions)

Variable	Total (*n*=117)	Bone lesions (*n*=51)	Soft tissue lesions (*n*=66)
Mean Age (y) [range, median]	33.7 [1-86, 31]	25.2 [9-71, 20]	40.3 [1-86, 41]
Sex (Male, Female)	55 (47%), 62 (53%)	30 (59%), 21 (41%)	25 (38%), 41 (62%)
Follow-up duration (m) [range, median]	22.2 [1-144, 10]	22.9 [1-120, 12]	21.7 [1-144, 8]
Anatomical location			
Hallux	13 (11%)	7 (14%)	6 (9%)
Lesser toe	55 (47%)	20 (39%)	35 (53%)
Midfoot	5 (4%)	3 (6%)	2 (3%)
Hindfoot and ankle	44 (38%)	21 (41%)	23 (35%)
Size (mm) [range, median]	23.5 [3-88, 19]	19.3 [3-58, 15]	26.8 [5-88, 23.5]
Surgery procedures		Marginal excision: 15 (29%)	Marginal excision: 57 (86%)
		Wide excision: 5 (10%)	Wide excision: 4 (6%)
		Curettage: 30 (59%)	Amputation: 4 (6%)
		Amputation: 1 (2%)	Intratumoral excision: 1 (2%)
Recurrence	10 (9%)	4 (8%)	6 (9%)
Complications	7 (6%)	Wound problem: 2 (4%)	Wound problem: 3 (5%)
		Fracture: 2 (4%)	
Benign lesion	102 (87%)	Total: 45 (88%)	Total: 57 (86%)
		Osteochondroma: 11	TGCT: 22 :11 (22%)
		Enchondroma: 9	Fibroma of tendon sheath: 7
		Solitary bone cyst: 8	Lipoma: 7
		Chondroblastoma: 4	Epidermoid cyst: 5
		Osteoid osteoma: 4	Ganglion:4
		Lipoma: 3	Angioleiomyoma: 3
		Chondrofibroma: 2	Hemangioma:3
		Ganglion: 2	Schwannoma: 3
		Others: 2	Others: 3
Intermediate malignancy	3 (3%)	Total: 1 (2%)	Total: 2 (3%)
		Desmoplastic fibroma: 1	Desmoid: 1
			Synovial chondromatosis with Cytological atypica: 1
Malignant lesion	12 (10%)	Total: 5 (10%)	Total: 7 (11%)
		Leiomyosarcoma:2	Synovial sarcoma: 2
		Osteosarcoma: 2	Alveolar rhabdomyosarcoma:1
		Metastasis:1	Alveolar soft part sarcoma:1
			Atypical fibrous histiocytoma:1
			Leiomyosarcoma:1
			Myxoinflammatory fibroblastic sarcoma: 1

*y* year, *m* month, *TGCT* Tenosynovial giant cell tumor

The tumor was located in the hallux in 13 patients (11%), in lesser toes in 55 cases (47%), in the midfoot in five cases (4%), and in the ankle/hindfoot in 44 cases (38%). An osteochondroma (22%), enchondroma (18%), and solitary bone cyst (16%) were the most frequently observed benign bone tumor and tumor-like lesions. Malignant bone tumors (5 cases: 4%) were osteosarcoma (2 cases), leiomyosarcoma (2 cases), and metastasis of lung squamous cell carcinoma (1 case). TGCT (33%) was the most frequent benign soft tissue lesion, whereas a variety of tumors (desmoid, synovial chondromatosis with cytological atypia, alveolar rhabdomyosarcoma, alveolar soft part sarcoma, atypical fibrous histiocytoma, leiomyosarcoma, myxoinflammatory fibroblastic sarcoma, and synovial sarcoma) were seen in intermediate malignant and malignant tumors.

For the benign bony lesions, 30 cases (59%) were treated with curettage and 15 cases (29%) underwent marginal excision, whereas primary foot amputation was performed in a patient with osteosarcoma. In four cases of tumors at the distal tibia (1 metastasis, 1 desmoplastic fibroma, and 2 leiomyosarcomas), ankle arthrodesis using retrograde intramedullary nail with pedicled fibula transfer was performed after wide resection of the distal tibia.

**Fig. 1:**
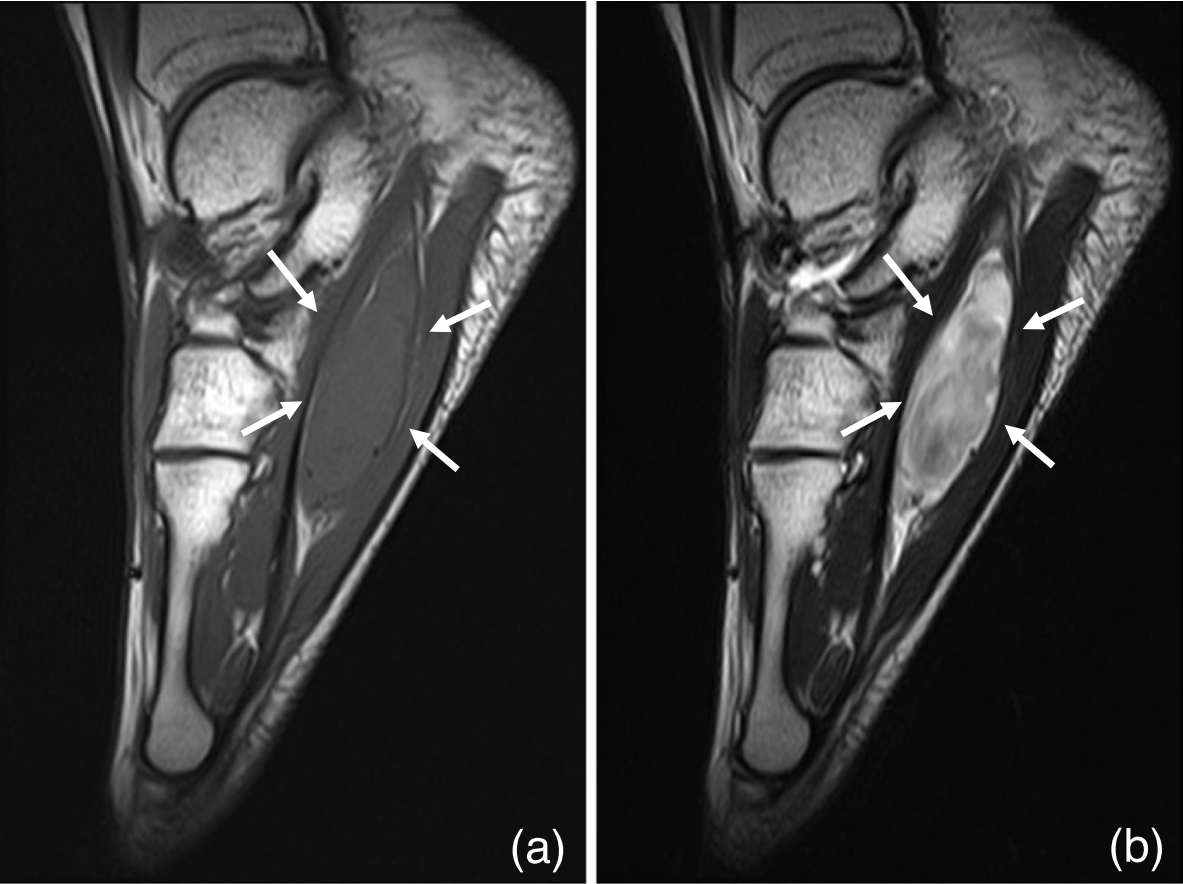
Synovial sarcoma occurred on planta. Sagittal T1 MRI image (**a**) and T2 MRI image (**b**) showing plantar lesion, presenting as synovial sarcoma. MRI, Magnetic resonance imaging

All 57 patients with benign soft tissue lesions (86%) underwent marginal resection, one with a desmoid tumor underwent intratumoral resection, and the one with synovial chondromatosis with cytological atypia got their foot amputated. A case of desmoid intermediate malignancy was managed with R1 resection, which was defined as a presence of microscopic residual tumor cells after resection [18]; it resulted in below-knee amputation due to recurrence. In malignant soft tissue tumors, four patients underwent wide resection, and three patients’ feet were amputated (Table 2). Two patients with sarcoma of the plantar fascia (1 alveolar rhabdomyosarcoma, 1 synovial sarcoma) were performed with the resection of the plantar fascia, flexor digitorum longus, flexor digitorum brevis, and quadratus plantae (Fig. 1). One patient with alveolar soft part sarcoma localized at medial foot subcutaneously was resected tumor and abductor halluces. The patient with subcutaneous leiomyosarcoma at the medial malleolar region was performed with resection of the tumor and a part of abductor hallucis. The recurrent tumors of bone were seen in four patients (8%) (1 chondrofibroma, 1 osteoid osteoma, 1 ganglion, and 1 leiomyosarcoma), and those of soft tissue tumors were observed in six patients (9%) (5 TGCTs and 1 desmoid). Postoperative complications like five wound problems (two in bony lesions, three in soft tissue) and two fractures in the cases of bone tumor were observed. Five wound problems, such as delayed wound healing and surgical site infection, had been cured by a bedside procedure and a course of antibiotics.

**Table 2:** Detailed clinical information of malignant and intermediate malignant tumors of the foot and ankle (*N* = 15)

Diagnosis		Age/ Sex	Anatomical location	Surgery procedures	Reconstruction	Adjuvant treatment	Follow-up (m)	Oncological Status	Total JSSF scale	
									pre operative	post operative
**Intermediate**										
Bone	Desmoplastic fibroma	24/M	Hindfoot	Wide excision	Ankle arthrodesis using intramedullary nail with pedicled fibula transfer	-	39	CDF	81	50
Soft tissue	Desmoid	20/M	Lesser toe	Intratumoral excision → Below-knee amputation	-	Chemo-therapy	45	AWD	9	-
	Synovial chondromatosis with cytological atypia	50/F	Lesser toe	Trans-metatarsal amputation	-	-	24	CDF	69	-
**Malignant**										
Bone	Metastasis from lung squamous cell carcinoma	68/M	Hindfoot	Wide excision	Ankle arthrodesis using intramedullary nail with pedicled fibula transfer	Chemo-therapy	6	DOD	2	-
	Leiomyosarcoma	34/M	Hindfoot	Wide excision → Below-knee amputation	Ankle arthrodesis using intramedullary nail with pedicled fibula transfer	Chemo-therapy	73	DOD	45	-
	Leiomyosarcoma	27/F	Hindfoot	Wide excision	Ankle arthrodesis using intramedullary nail with pedicled fibula transfer	Chemo-therapy	43	AWD	58	33
	Osteosarcoma	20/M	Lesser toe	MTP disarticulation	-	Chemo-therapy	82	CDF	59	-
	Osteosarcoma	18/M	Hindfoot	Wide excision	Ankle arthrodesis using screws	Chemo-therapy	70	CDF	75	59
Soft tissue	Alveolar rhabdomyosarcoma	18/F	Lesser toe	Wide excision	Tendon transplant	Chemo-therapy, Radiation	18	CDF	70	70
	Alveolar soft part sarcoma	14/M	Hindfoot	Wide excision	-	-	36	CDF	84	74
	Atypical fibrous histiocytoma	57/F	Lesser toe	Below-knee amputation	-	-	48	CDF	58	-
	Leiomyosarcoma	73/F	Hindfoot	Wide excision	Pedicled flap and skin graft	-	48	CDF	100	100
	Myxoinflammatory fibroblastic sarcoma	62/F	Hallux	Amputation at 1st metatarsal	-	-	74	CDF	87	-
	Synovial sarcoma	86/F	Lesser toe	Chopart disarticulation	-	-	11	DOD	90	-
	Synovial sarcoma	14/F	Lesser toe	Wide excision	Tendon transplant	Chemo-therapy	53	CDF	74	90

*M* Male, *F* Female, *m* month, *MTP* Metatarsophalangeal joint, *CDF* continuously disease free, *AWD* alive with disease, *DOD* dead of disease

### Clinical course in the case of intermediate malignant and malignant tumor

Table 2 presents the clinical course of 3 cases with intermediate malignancy and 12 patients with malignant tumors. Total 7 patients (47%) of intermediate and malignancy, including desmoid, synovial chondromatosis with cytological atypia, leiomyosarcoma of bone, osteosarcoma, atypical fibrous histiocytoma, myxoinflammatory fibroblastic sarcoma, and synovial sarcoma, had been amputated of the foot or below knee finally. Postoperative patients with 3 malignancy (20%), including metastasis, synovial sarcoma, and leiomyosarcoma of bone, has resulted in death of disease. Total 10 patients (67%) have continuously lived with disease free after resection of the tumor, and 4 of whom resulted in amputation. The patient with leiomyosarcoma underwent wide excision and reconstruction by pedicle flap and skin graft. Plantar malignant tumors, namely alveolar rhabdomyosarcoma and synovial sarcoma (Fig. 1), were managed with wide excision followed by tendon transplant. The postoperative JSSF scores of limb-sparing patients with CDF were significantly unchanged (mean, 74; range, 50–100) (*p* = 0.17), compared with preoperative JSSF scores (mean, 81; range, 70–100).

### Clinical outcomes

**Fig. 2:**
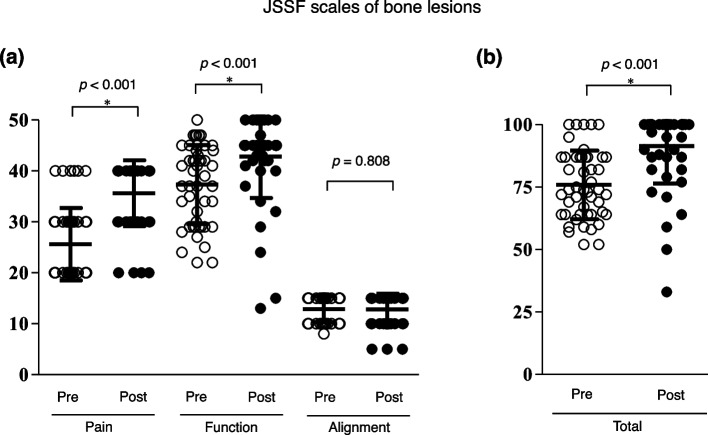
Preoperative and postoperative JSSF scale of the bone lesions in each subcategory (**a**) and total scale (**b**). Black solid line indicated the mean and standard deviation. Paired t-test was performed, respectively (*p*-value was shown above graph, each). JSSF, Japanese Society of Surgery of the Foot

The distribution of preoperative and postoperative JSSF scores for tumor and tumor-like lesions of the bone and soft tissue are presented in Figs. 2 (2a and 2b) and 3 (3a and 3b), respectively. The mean preoperative pain scores for bone and soft tissue lesions were 25.6 ± 7.0 and 31.0 ± 8.5, respectively, while there was a significant improvement in the mean postoperative pain scores to 35.6 ± 6.5 and 35.2 ± 7.2, respectively (*p* < 0.001, *p* < 0.001). The average postoperative function scores for bone and soft tissue lesions improved significantly from preoperative values (bone lesion: 37.4 ± 7.8 to 42.8 ± 8.1; soft tissue lesion: 40.7 ± 6.8 to 43.2 ± 5.5) (*p* < 0.001, *p* = 0.004). Consequently, the total postoperative JSSF scale scores indicated a significant improvement for both types of lesions (bone lesion: 75.9 ± 13.7 to 91.4 ± 14.9; soft tissue lesions: 84.7 ± 14.8 to 91.9 ± 12.5) (*p* < 0.001, *p* < 0.001).

**Fig. 3:**
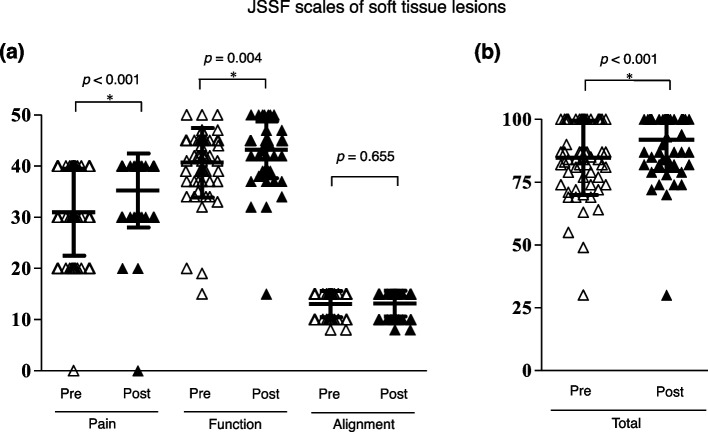
Preoperative and postoperative JSSF scale of the soft tissue lesions in each subcategory (**a**) and total scale (**b**). Black solid line indicated the mean and standard deviation. Paired t-test was performed, respectively (*p*-value was shown above graph, each)

**Fig. 4:**
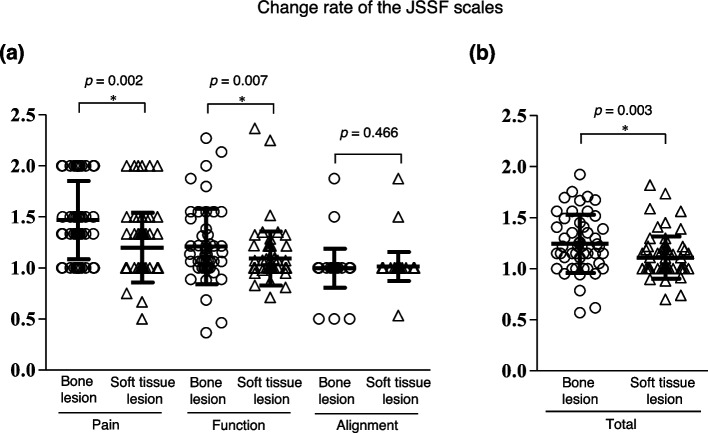
The change rate of comparison between bone and soft tissue lesions using the JSSF scale from preoperative to postoperative in each subcategory (**a**) and total scale (**b**). The black solid line indicated the mean and standard deviation. Mann–Whitney U test was performed, respectively (*p*-value was shown above graph, each)

The improvement rates for preoperative and postoperative JSSF scale scores were also analyzed. The improvement rate in pain and function scores in bony lesions was significantly greater than for the soft tissue lesions (*p* = 0.002, *p* = 0.007) (Fig. 4a and b).

## Discussion

This study retrospectively evaluated the postoperative clinical outcomes in patients with tumors and tumor-like lesions of the foot and ankle. As reported earlier, tumors of the foot and ankle form a small proportion of musculoskeletal tumors (5%–10%), the incidence of malignant tumors being much lower than the benign ones [8, 9, 18–21]. Comparable to the existing literature describing the distribution pattern of tumors in this region [8, 9], bone lesions in our study were significantly higher in young males than soft tissue lesions. Osteochondroma (22%), enchondroma (18%), and bone cysts (16%) account for more than half of benign bone tumors. Benign bone lesions were treated with marginal excision or curettage. TGCT (33%) was the most common benign soft tissue lesion and was treated with marginal excision. As a previous study reported that TGCT, especially the diffused-type, was associated with high recurrence (33%) [22, 23], 5 of our cases with diffused-type TGCT (23%) demonstrated a recurrent lesion. Ozdemir et al. reported a recurrence rate of 6.6% for tumors of the foot and ankle [1], we observed a similar recurrence rate of 8.5% (7.8% for bone lesions; 9.1% for soft tissue lesions).

The 5-year survival rate for malignant tumors of the foot and ankle is reported to be 80%, with 44–88% requiring amputation [2, 24, 25]. In this study, as in previous reports, 20% of patients with malignant or intermediate malignancy had died from diseases, and 53% had undergone amputation. Usually, to fulfill the oncological goal of treatment, wide resection and reconstruction are warranted; however, when complete resection of the tumor is difficult or there is a high risk of local recurrence after resection, amputation of the foot or the leg is recommended [26]. Malignant bone tumors of the forefoot should be treated locally with amputation at a level appropriate for the proximal extension of the tumor [27]; accordingly, in three of our cases, amputations at the metatarsal and metatarsophalangeal disarticulation were performed in the first surgery, and neither of them reported recurrence. Additionally, postoperative complications, especially surgical site infection, occur in 2 to 5.1% of surgeries of the foot and ankle [28–30]; as mentioned above, five of our patients (4.3%) developed wound problems, such as delayed wound healing and surgical site infection, which is comparable to previous reports.

Due to the small-sized compartments of the foot and ankle region, it is easy to localize a mass and pain in this area. It is reported that the main complaints associated with tumors of the foot ankle were noticeable mass and pain [6, 31, 32]. The present study confirmed that the pain improved significantly after the surgical treatment and that patients with bony lesions suffered from preoperative pain more than those with soft tissue lesions (Fig. 4). Since a bone lesion may be associated with pathological microfractures, the postoperative JSSF pain scores for bony lesions were significantly improved owing to the healing of the fracture. In this study, the improvement in pain and function was relatively small despite statistically significant change (*p* < 0.01). However, previous evidence [33, 34] have demonstrated that the minimal clinically important difference of the total AOFAS score was 8–10 points, indicating that the total JSSF scale scores in our study might show a significant improvement statistically.

Hitherto, there have been some epidemiological studies about tumors of this region [1–6, 8, 9], which have demonstrated the diagnosis and distribution patterns of tumors of foot and ankle, and predicted factors for the grade of malignancy. However, none of them have reported the postoperative clinical and functional outcomes. Functional evaluation after surgical treatment of musculoskeletal tumors is usually done by the Musculoskeletal Tumor Society (MSTS) scoring system [35]; however, the MSTS system is a scale for determining the physical and mental health status of patients with tumor at the limb and has difficulty evaluating the specific function of the foot and ankle. Therefore, the JSSF scale was used to evaluate the postoperative function of the foot and ankle in this study. We found that the pain and function were significantly improved postoperatively, suggesting that resection of the mass and disappearance of the pain may have led to better postoperative clinical outcomes in terms of walking, activities of daily living, and putting on their shoes. After limb-sparing surgery for malignancy of the foot and ankle, preoperative and postoperative JSSF scale scores have shown no significant change.

This study has several limitations. First, we used a retrospective study design for analyzing clinical data. Second, we investigated only a small number of patients from a single institution without a control group; however, it was logistically not possible to have a strict control group since tumors of the foot and ankle are extremely rare [1–3]. Therefore, this study included various kinds of tumors and tumor-like lesions of the foot and ankle, and was designed as a retrospective study. In addition, since the distribution of tumors and tumor-like lesions of the foot and ankle in this study were similar to previous studies [8, 9], the sufficient number of patients at a single institution might be analyzed. The strong point of a single institution study is that Kyushu university hospital, which is a musculoskeletal tumor referral center, could determine the consistent management of treatment. Third, only patients who underwent excision surgery were included in the study, and we excluded patients with benign lesions with conservative treatment (e.g., hemangioma, ganglion, etc.). These patients with conservative treatment had been unchanged in pattern and size after several MRI exams or biopsies. Additionally, because most of the patients with conservative treatment had slight symptoms and less dysfunction, we could exclude them from this study. Fourth, the follow-up duration in this study has a huge level of variance, 1–144 months. As previous studies [10, 25] have also shown the various variance, some patients with benign lesions might quit follow-up shortly due to the improvement of pain and function of the affected limb. Actually, in our patients ended follow-up within 1 year, the mean final JSSF scale scores were 37.8 ± 5.3 (pain), 44.8 ± 3.2 (function), 14.1 ± 2.0 (alignment), and 96.6 ± 7.9 (total), each. These good outcomes may be unchanged, suggesting that final JSSF scale scores could be compared in patients with short or long-term follow-up. Fifth, the follow-up duration of some patients with malignant tumor was relatively short (range: 18–82 months). Although we have the necessity to careful follow-up continuously, all patients had been continuous disease free at the final visit. Finally, the JSSF scale was inappropriate to assess the amputated legs, so we excluded them in the analysis.

## Conclusions

This study elucidated the postoperative clinical outcomes in patients with tumors and tumor-like lesions of the foot and ankle which are relatively rare entities. Malignant tumors of this region comprise a further smaller proportion (10% in this study). The clinical outcomes as evaluated using the JSSF scale revealed improvements in pain and function postoperatively, more so with bony lesions than soft tissue lesions due to the significant improvement in pain.

### Take home message

Tumor and tumor-like lesions of the foot and ankle are rare, with malignant tumors showing lower incidence. Bone lesions showed greater improvement in pain and function than soft tissue lesions. Postoperative clinical results, including malignancy, show good clinical and functional outcomes as measured by the JSSF scale.

## Data Availability

The datasets generated during and analyzed during the current study are not publicly available due to their containing information that could compromise the privacy of research participants but are available from the corresponding author on reasonable request.
